# Is kallikrein-8 a blood biomarker for detecting amnestic mild cognitive impairment? Results of the population-based Heinz Nixdorf Recall study

**DOI:** 10.1186/s13195-021-00945-x

**Published:** 2021-12-20

**Authors:** Sara Schramm, Martha Jokisch, Karl-Heinz Jöckel, Arne Herring, Kathy Keyvani

**Affiliations:** 1 Institute of Medical Informatics, Biometry and Epidemiology, University Hospital Essen, University of Duisburg-Essen, Hufelandstr. 55, 45122 Essen, Germany; 2 Department of Neurology, University Hospital Essen, University of Duisburg-Essen, Essen, Germany; 3 Institute of Neuropathology, University Hospital Essen, University of Duisburg-Essen, Essen, Germany

**Keywords:** Amnestic mild cognitive impairment, MCI, Kallikrein-8, KLK8, Neuropsin, Alzheimer’s disease, Dementia, Biomarker, Heinz Nixdorf Recall study, Neurodegeneration

## Abstract

**Background:**

Kallikrein-8 (KLK8) might be an early blood-biomarker of Alzheimer’s disease (AD). We examined whether blood KLK8 is elevated in persons with amnestic mild cognitive impairment (aMCI) which is a precursor of AD, compared to cognitively unimpaired (CU) controls.

**Methods:**

Forty cases and 80 controls, matched by sex and age (± 3years), were participants of the longitudinal population-based Heinz Nixdorf Recall study (baseline: 2000–2003). Standardized cognitive performance was assessed 5 (T1) and 10 years after baseline (T2). Cases were CU at T1 and had incidental aMCI at T2. Controls were CU at T1 and T2. Blood KLK8 was measured at T2. Using multiple logistic regression the association between KLK8 in cases vs. controls was investigated by estimating odds ratios (OR) and 95% confidence intervals (95%CI), adjusted for inter-assay variability and freezing duration. Using receiver operating characteristic (ROC) analysis, the diagnostic accuracy of KLK8 was determined by estimating the area under the curve (AUC) and 95%CI (adjusted for inter-assay variability, freezing duration, age, sex).

**Results:**

Thirty-seven participants with aMCI vs. 72 CU (36.7%women, 71.0±8.0 (mean±SD) years) had valid KLK8 measurements. Mean KLK8 was higher in cases than in controls (911.6±619.8 pg/ml vs.783.1±633.0 pg/ml). Fully adjusted, a KLK8 increase of 500pg/ml was associated with a 2.68 (1.05–6.84) higher chance of having aMCI compared to being CU. With an AUC of 0.92 (0.86–0.97), blood KLK8 was a strong discriminator for aMCI and CU.

**Conclusion:**

This is the first population-based study to demonstrate the potential clinical utility of blood KLK8 as a biomarker for incipient AD.

**Supplementary Information:**

The online version contains supplementary material available at 10.1186/s13195-021-00945-x.

## Background

Alzheimer’s disease (AD) is a neurodegenerative disease and the most common cause of dementia. AD starts to damage the brain 20 years or more before any clinical symptoms appear [1, 2]. To slow or stop the progression of AD, future treatments have to be administered in the very early, ideally antecedent stages of the disease continuum. The currently available biomarker for AD (beta-amyloid (Aβ, A), tau (T), and neurodegeneration (N), (ATN)) are measured in cerebrospinal fluid (CSF) or by neuroimaging techniques like positron emission tomography and magnetic resonance imaging [3]. As the measurement of these biomarkers is expensive and invasive, it is not applicable in primary care settings. Thus, less-invasive and less-expensive blood-based biomarkers of tau and Aβ have been developed and ultrasensitive immunoassay techniques allow to measure even small amounts of brain-specific proteins in blood samples [4]. Other biomarkers of AD are required in addition to the standard ATN marker.

The extracellular serine protease kallikrein-8 (KLK8, alias neuropsin) is a well-known, dose-dependent modulator of neuroplasticity and memory [5–8]. It unfolds its effects in part by processing the substrates neuregulin-1 [9], neuronal cell adhesion molecule L1 [10], fibronectin [11], and ephrin receptor B2 [12] and thereby regulates different neuroplasticity associated signaling pathways [7, 13–15]. Our knowledge about the role of KLK8 in pathophysiological processes is, however, very limited. KLK8 is thought to be associated with epilepsy [16], depression [17], and multiple sclerosis [18], but until recently, almost nothing was known in the context of AD. Our lab was the first to show pathologically high levels of KLK8 mRNA and protein in different regions of transgenic murine [19] and AD-affected human brain [19, 20] long before the clinical signs of disease appear. This exceedingly early (in mice prior to onset of amyloid pathology; in patients in CERAD A/Braak I-II stage) and multifocal rise of KLK8 [19] is suggestive of a causal role in the cascade of events leading to AD. This hypothesis is supported further by the fact that even a short-term inhibition of this enzyme by an anti-KLK8-antibody [19, 21] or its long-term downregulation by genetic knockdown [22], exerted considerable therapeutic effect in transgenic mice. It impedes amyloidogenic amyloid-precursor-protein processing, facilitates Aβ clearance, boostes autophagy, reduces Aβ load and tau pathology, enhances neuroplasticity, improves memory, and unfolds anxiolytic effects [19, 21, 22]. Furthermore, KLK8 levels were also elevated in blood and CSF of patients with MCI due to AD and early AD [23]. It has been shown that the diagnostic accuracy of CSF KLK8 was as good as that of core CSF biomarkers (Aβ, phosphorylated tau and total tau) for AD and in case of MCI even superior to CSF Aβ_42_. Blood KLK8 was a similarly strong discriminator for MCI due to AD but slightly weaker for AD [23]. Thus, KLK8 might not only be a therapeutic target but also a very early biomarker of AD.

The aim of the present case-control study was to examine whether blood KLK8 is elevated in participants with amnestic MCI (aMCI) compared to cognitively unimpaired (CU) participants in a population-based sample.

## Methods

### Study design and study population

The analysis is based on data of the longitudinal population-based Heinz Nixdorf Recall (Risk Factors, Evaluation of Coronary Calcification, and Lifestyle) study (HNR study). Details of the study design and cohort have been described previously [24]. Briefly, participants for this study were randomly selected inhabitants of the Ruhr area living in Essen (589,676 residents), Bochum (371,582 residents), and Mülheim/Ruhr (172,759 residents). Baseline examination from 2000 to 2003 included 4814 men and women age 45–75 years with an overall recruitment efficacy proportion of 55.8% [25]. Participants received annual questionnaires and follow-up examinations after 5 years (T1) and 10 years (T2).

**Fig. 1:**
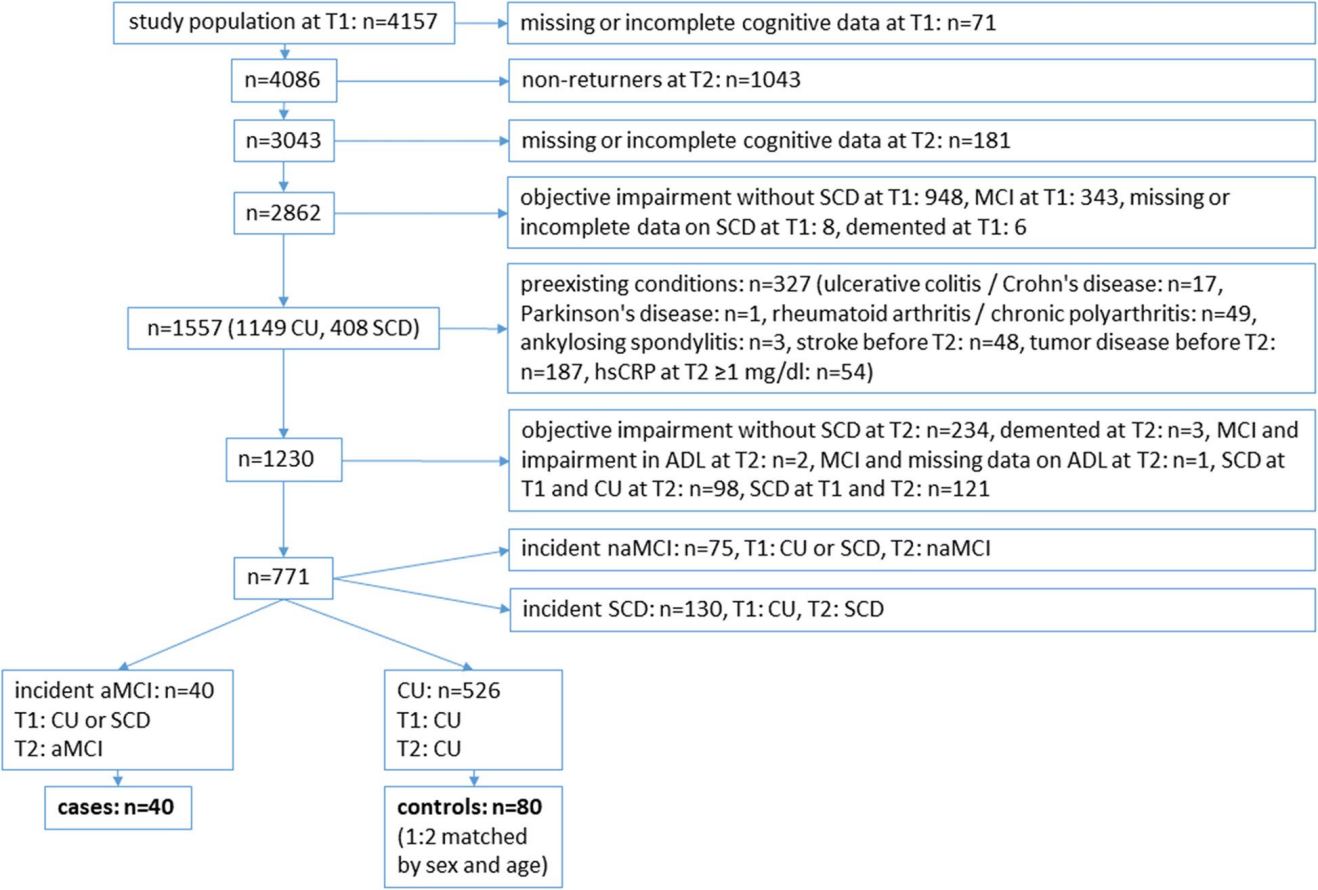
Flow chart of the study population. Legend: T1, first follow-up visit; T2, second follow-up visit; CU, cognitively unimpaired; aMCI, amnestic mild cognitive impairment; SCD, subjective cognitive decline; naMCI, non-amnestic MCI; ADL, activities of daily living; hsCRP, high-sensitivity C-reactive protein

In our case-control study, we included participants which were CU or had subjective cognitive decline (SCD) at T1 and had an incidental aMCI diagnosis at T2 (cases, for definition of aMCI see below). Controls had to be CU at T1 and T2. At least to T2, cases and controls were free of the secondary diseases mentioned below. Two controls were assigned to each case, matched by sex and age (± 3 years). Supplementary material 1 shows the results of the matched case-control power analysis. Figure 1 shows the flow-chart of the study population.

All participants provided written informed consent. The study was approved by the University of Duisburg-Essen Institutional Review board and followed established guidelines of good epidemiological practice.

### Survey of secondary diseases

Participants received standardized computer-assisted interviews and were asked whether they have/had any of the following conditions: ulcerative colitis, Crohn’s disease, Parkinson’s disease, rheumatoid arthritis, chronic polyarthritis, ankylosing spondylitis, stroke, or tumor disease before T2. Additionally, they were asked in yearly postal follow-up questionnaires about having stroke or cancer. Participants who denied those diseases until T2 and had no high-sensitivity C-reactive protein (hsCRP) ≥ 1 mg/dl (reference value: 0.3 mg/dl) at T2 were classified as free of those diseases and were included into our study. We decided to exclude participants with Parkinson’s disease, stroke, and tumor disease to exclude cognitive impairment due to those diseases. As there is some evidence of an association between KLK8 and inflammation [26], we also decided to exclude participants with signs of inflammation.

### Measurement of KLK8

Details of the KLK8 measurements and technical specifications of the utilized KLK8 enzyme-linked immunosorbent assay (ELISA) kit have been described previously [23]. Briefly, KLK8 measurements in T2 blood were performed in the central reference laboratory at the Institute of Neuropathology, University of Duisburg-Essen. Experimenters were blinded to participants’ diagnoses. KLK8 levels were measured in duplicate using a commercially obtained ELISA kit (#EK0819, Boster Biological Technology, Pleasanton, CA, USA) following the manufacturer’s instructions. Kits came from two lots. Experimenter 1 measured in February 2020 and experimenter 2 and 3 in March 2021. Serum samples were diluted 1:2 in sample buffer. Agreement between the two measurements was assessed graphically using a scatter plot and a Bland-Altman plot (Supplementary Figs. S1 and S2). Only two subjects had mean KLK8 values that were outside the 95% confidence interval, implicating a high reliability of KLK8 measurements.

### Cognitive performance

A standardized cognitive performance assessment in the HNR study was introduced at T1 and was extended for T2. Details of the cognitive performance assessment have been described previously [27, 28]. In brief, cognitive performance at T1 was assessed with five subtests. These included established measures of immediate and delayed verbal memory (eight word list [29], speed of processing/problem solving (Labyrinth test [29]), verbal fluency (semantic category “animals” [30]), and visuo-spatial ability (clock-drawing test [31]). For a detailed assessment description, see Wege et al. [32]. The MCI diagnosis at T1 based on these five subtests was validated in a previous study. The short cognitive performance assessment showed a good accuracy compared to a detailed neuropsychological and neurological examination (area under the curve = 0.82, 95% confidence interval = 0.78–0.85).

Regarding T2, the cognitive performance assessment was extended by Trail Making Test A (TMT A9), Trail Making Test B (TMT B [33]), and a short version of the Stroop task named Color-word test [29] (card 1: word reading; card 2: color naming; card 3: color-word interference condition; card 3 minus card 2: interference performance [34]).

For the five subtests that have already been administered at T1, z-transformation of the raw data at T2 using our own defined norm-data from T1 was performed: Raw data were z-transformed based on the mean and SD of the appropriate age- and education group at T1 (age: 50–59 years, 60–69 years, and ≥70 years; education: ≤10 years, 11–13 years, and ≥14 years) [28]. For the subtests of the extended cognitive performance, z-transformation was based on the same education groups and the following three age groups from T2: 55–64 years, 65–74 years, and ≥75 years. Except for the clock-drawing test, the age- and education-adjusted test scores were scaled to have a mean of 10 and a standard deviation (SD) of 3 [28, 35]. The administered tests were grouped into four domains: (1) attention—Trail Making Test A, Color-word test card 1 and card 2; (2) executive function—Trail Making Test B, Labyrinth test, Color-word test interference performance, verbal fluency; (3) verbal memory—eight word list immediate and delayed recall; and (4) visuoconstruction—clock-drawing test [28]. Within each domain, newly scaled scores of the tests were added. To account for the differing numbers of tests in each domain, domain scores were then scaled to have a mean of 10 and a SD of 3. Cognitive impairment was defined as a performance of more than one SD below the mean (≤7) in at least one total domain score of the domains attention, executive function, verbal memory, or as a score of ≥3 in visuoconstruction [28, 31]. We have used one SD below the mean to rate a domain as impaired as suggested by Albert et al. [36] for the core clinical criteria of MCI.

The diagnosis of dementia was based on DSM-IV dementia diagnosis criteria [37] requiring cognitive impairment to be “significant” and affect activities of daily living. In our study, the “significance” of cognitive impairment was defined by two standard deviations below the age- and education-adjusted mean as our standard—a criterion that is now part of the DSM-5 definition of major neurocognitive disorder [38]. Further, dementia diagnosis was defined as a previous physician’s diagnosis of dementia or taking cholinesterase inhibitors (anatomic-therapeutic-chemical classification issued by the World Health Organization (WHO), code: N06DA) [39] or other anti-dementia drugs (N06DX). We excluded six participants with dementia at T1 and three at T2.

### Definition of cases and controls

Cases were participants with an aMCI diagnosis based on meeting all of the following published Winblad et al. aMCI criteria [40]: (1) cognitive impairment in the verbal memory domain (with or without impairments in other above named three domains); (2) subjective cognitive decline; (3) normal functional abilities and daily activities; and (4) no dementia diagnosis (definition see above). This definition is equivalent with the core clinical criteria of “MCI due to AD” according to Albert et al. [36] in the absence of further biomarker information.

To examine incident aMCI, participants with MCI, dementia or participants who fulfilled criteria 1, 3, and 4 of the MCI diagnosis without criterion 2 at T1 were excluded (objective impairment without SCD). Controls were CU at T1 and T2. Thus, their cognitive performance was within the age- and education- adjusted range in all domains, and they did not report subjective cognitive decline at T1 or T2.

### Apolipoprotein E

To discriminate between the APOE alleles ε2, ε3, and ε4, Cardio-MetaboChip BeadArrays were used for genotyping of two single-nucleotide polymorphisms (rs7412 and rs429358). Participants defined as APOE ε4 positive had at least one allele 4 (2/4, 3/4, 4/4). All other participants were defined as APOE ε4 negative [28].

### Assessment of covariates

Body mass index at T2 (BMI in kg/m^2^) was calculated from measured height and weight. Education until T2 was classified according to the International Standard Classification of Education (ISCED-97) as total years of formal education, combining school and vocational training [41]. ‘Current smoking’ at T2 was defined as a history of cigarette smoking during the past year, ‘Past smoking’ as quitting smoking more than a year ago, otherwise no. ‘Sports’ at T2 was defined as ‘yes’ when practiced one or more sports in the last 4 weeks prior to the interview, otherwise no. Blood pressure categories were defined according to the Joint National Committee 7 guidelines [42]. ‘Hypertension’ at T2 was defined as stage 1 or 2, otherwise no. Current depressive symptoms at T2 were assed using the German 15-item short form of the Center for Epidemiologic Studies Depression Scale (CES-D). The cut-off point for ‘elevated depressive symptoms’ was ≥18 [43]. ‘Diabetes mellitus’ at T2 was defined present when participants reported a diagnosis of diabetes mellitus, or used antidiabetic medication, or had an elevated fasting serum glucose of ≥ 200 mg/dl.

### Statistical analysis

Descriptive statistics were performed. To compare cases and controls *p*-values were estimated with Wilcoxon two sample test (continues variables, not normally distributed) or chi-square test (nominal variables). Box plots were created to show the distribution of mean KLK8 by strata according to cognitive status. Box plots were created to show the distribution of the predictive values of KLK8 according to cognitive status, adjusted for cognitive status, freezing duration, age, sex, and inter-experimenter variability, which should be understood as a proxy for the inter-assay variability (and hereinafter referred to as ‘inter-assay variability’). Using conditional multiple logistic regression, the association between KLK8 and aMCI compared to CU was determined by estimating odds ratios (OR) and 95% confidence intervals (95%CI), adjusted for inter-assay variability and freezing duration. The diagnostic performance of KLK8 was determined using receiver operating characteristic (ROC) analyses, adjusted for inter-assay variability, freezing duration, age, and sex. All analyses were performed using SAS 9.4 (Statistical Analysis System Corp., Cary, NC, USA).

## Results

Our study population comprises 40 cases with incident aMCI with frozen blood samples at T2, which were free of the above-mentioned diseases at T2. *N*=526 participants were CU at T1 and T2 and free of the above-mentioned diseases at T2. After matching for sex and age ±3 years, we had 80 controls with frozen blood samples at T2 (Fig. 1). In three cases and eight controls, KLK8 was below detection threshold.

Table 1 shows the characteristics of the study population according to the cognitive status. Out of 109 participants, 36.7% were women and the mean age was 71.0±8.0 years. The 37 participants with aMCI had a higher KLK8 mean value compared to the 72 CU participants (911.6±619.8 pg/ml vs. 783.1±633.0 pg/ml). Participants with aMCI were more often APOE ε4 positive compared to CU participants (48.6 vs. 26.4%) and had lower *z* scores in all four domains. According to the cut-off (CES-D≥18) 16% of the participants with aMCI had depression, and CU were free of depression. At T1, only four participants with aMCI had depression, and CU at T1 also were free of depression (data not shown). The mean freezing duration was lower in participants with aMCI compared to CU participants (7.5±1.0 years vs. 8.3±0.7). There were no major differences between cases and controls regarding the other variables (age, BMI, education, smoking status, sports, hypertension, and diabetes mellitus).

**Table 1:** Characteristics of the study population at T2, *n*(%), mean±SD

	aMCI ***n***=37	CU ***n***=72	Total ***n***=109	***p***-values
**KLK8, pg/ml**	911.6±619.8	783.1±633.0	826.7±628.7	0.24
**Sex**	23 (62.2)		69 (63.3)	0.86
**Men**		46 (63.9)		
**Women**	14 (37.8)	26 (36.1)	40 (36.7)	
**Age, years**	70.9±8.2	71.0±7.9	71.0±8.0	0.97
**BMI, kg/m**^**2**^	29.2±5.3	28.1±3.3	28.5±4.1	0.36
**Years of education**	14.0±2.4	14.7±2.4	14.5±2.4	0.20
**Smoking status**	16 (43.2)		49 (45.0)	0.82
**Never**		33 (45.8)		
**Past**	17 (45.9)	29 (40.3)	46 (42.2)	
**Current**	4 (10.8)	10 (13.9)	14 (12.8)	
**Sports**	18 (48.6)		49 (45.0)	0.58
**No**		31 (43.1)		
**Yes**	19 (51.4)	41 (56.9)	60 (55.0)	
**APOE ε4 positive**				0.01
**No**	17 (45.9)	52 (72.2)	69 (63.3)	
**Yes**	18 (48.6)	19 (26.4)	37 (33.9)	
**Missing**	2 (5.4)	1 (1.4)	3 (2.8)	
***z*** **score attention**	9.5±4.2	11.3±1.8	10.7±2.9	0.02
***z*** **score executive function**	8.7±3.7	11.3±1.8	10.4±2.9	<.01
***z*** **score verbal memory**	5.9±1.1	11.2±2.3	9.4±3.2	<.01
**z score visuoconstruction**	10.3±2.7	11.2±1.9	10.9±2.3	0.11
**Depression**^a^	6 (16.2)	0	6 (5.5)	<.01
**Missing**	2 (5.4)	3 (4.2)	5	
**Hypertension**				0.82
**No**	27 (73.0)	54 (75.0)	81 (74.3)	
**Yes**	10 (27.0)	18 (25.0)	28 (25.7)	
**Diabetes mellitus**				0.54
**No**	32 (86.5)	59 (81.9)	91 (83.5)	
**Yes**	5 (13.5)	13 (18.1)	18 (16.5)	
**Freezing duration, years**	7.5±1.0	8.3±0.7	8.0±0.9	<.01

*T2* ten-year follow-up, *SD* standard deviation, *aMCI* amnestic mild cognitive impairement, *CU* cognitive unimpaired, *KLK8* kallikrein 8, *BMI* body mass index, *APOE ε4* Apolipoprotein E ε4

^a^ Elevated depressive symptoms (CES-D≥18)

**Fig. 2:**
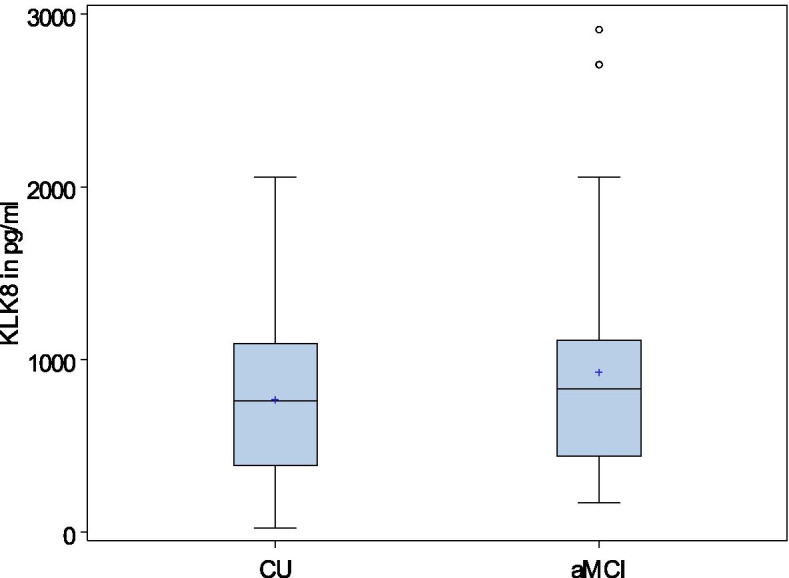
Distribution of mean KLK8 in pg/ml according to cognitive status and strata. Legend: KLK8, kallikrein 8; CU, cognitive unimpaired; aMCI, amnestic mild cognitive impairment. A strata contains one case and two controls according to our matched case-control design

**Fig. 3:**
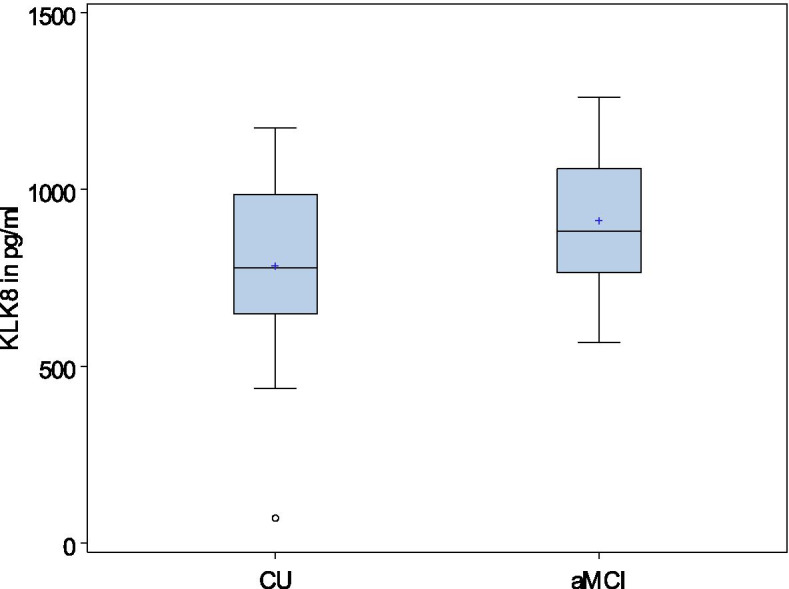
Distribution of predictive values of KLK8 (pv_KLK8) in pg/ml according to the cognitive status, adjusted for inter-assay variability, freezing duration, age, and sex. Legend: KLK8, kallikrein 8; CU, cognitive unimpaired; aMCI, amnestic mild cognitive impairment

Differences in KLK8 values in groups are also shown in box plots in Fig. 2 (mean values according to strata) and Fig. 3 (adjusted predictive values of KLK8). After adjustment KLK8 levels are generally higher in aMCI than in CU.

Table 2 shows the results of the conditional logistic regression analyses to estimate the association between KLK8 and cognitive status. Fully adjusted, a KLK8 increase of 500pg/ml was associated with a 2.68-fold (95%CI: 1.05–6.84) higher chance of having aMCI compared to being CU. When excluding participants with elevated depression (CES-D≥18) form our analyses, our results on the association of KLK8 and aMCI did not change (Supplementary material Table S1). Using the same and less stringent aMCI diagnostic criteria of T1 also at T2 did not affect the degree of KLK8 association with aMCI (Supplementary material Table S2).

**Table 2:** Association between KLK8 and cognitive status (aMCI vs. CU)

	OR	95%CI		OR	95%CI	
**Per 500 pg/ml KLK8**	1.113	0.821	1.511	2.683	1.053	6.835
**Experimenter 2 vs. 3**^a^				0.031	0.002	0.577
**Experimenter 1 vs. 3**^a^				5.916	0.897	39.033
**Freezing duration, years**				0.927	0.842	1.020

Age and sex are taken into account by matching for them

*aMCI* amnestic mild cognitive impairment, *CU* cognitive unimpaired, *OR* odds ratio, *95%CI* 95%-confidence interval, *KLK8* kallikrein 8

^a^ We adjusted for experimenter as a proxy for the inter-assay variability

**Fig. 4:**
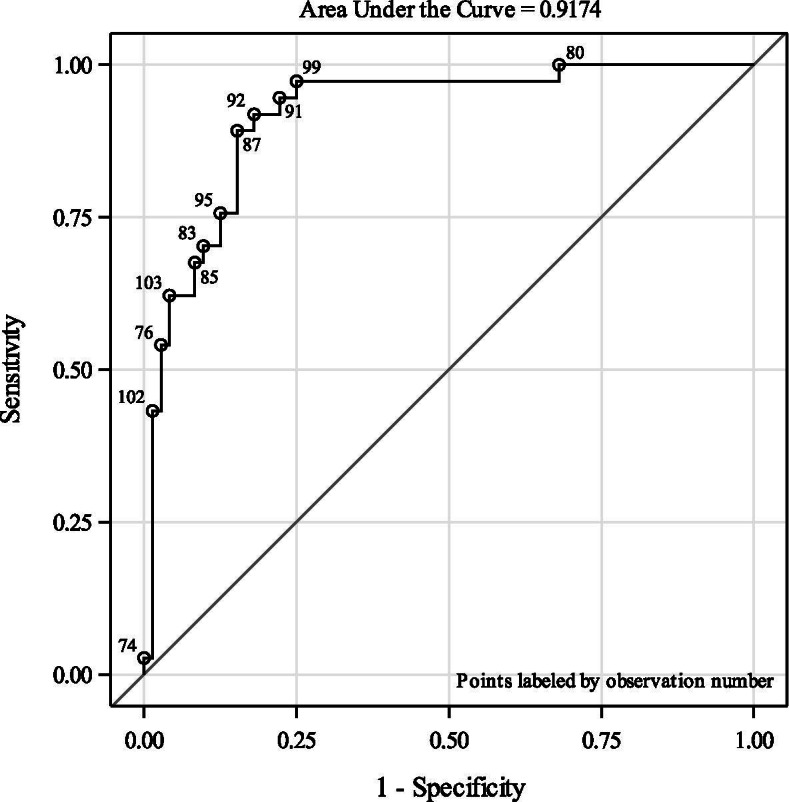
Diagnostic performance of KLK8 according to receiver operating characteristic (ROC) analyses, adjusted for inter-assay variability, freezing duration, age, and sex

Figure 4 shows the ROC curve after adjustment for inter-assay variability and freezing duration. The area under the curve (AUC) is 0.92 (95%CI: 0.86–0.97). The crude ROC curve is presented in Supplemental material Fig. S3.

## Discussion

Our study is the first population-based case-control study to investigate whether KLK8 is a suitable blood-based biomarker for the diagnosis of incident aMCI, a precursor of AD. We found an increase of blood KLK8 of 500 pg/ml to be associated with a 2.68-fold increased odd of an aMCI diagnosis in comparison to cognitively healthy participants. The diagnostic performance of blood KLK8 for aMCI was very good with an AUC of 0.92.

Our results are quite in line with the recently published data from Teuber-Hanselmann et al. [23] that showed the diagnostic accuracy of CSF KLK8 to be as good as accuracy of core CSF biomarkers, i.e., Aβ42/Aβ40 or phosphorylated tau with blood KLK8 being a similarly strong discriminator for MCI (AUC: 0.94; 0.86–1.00) as in the present study. However, the KLK8 cut-off point of 1121 pg/ml in that study was associated with a 130fold increased odd of MCI due to AD (95% CI: 15–1100) in comparison to controls. When applying the same cut-off value to our sample, we found a 2.62fold increased odd of aMCI (0.61–11.33; data not shown). This is most likely due to methodological differences between both studies. Teuber-Hanselmann et al. examined participants form a clinical setting not from a population-based sample. It is more likely that these participants were more severely affected by their cognitive symptoms and were hospitalized to evaluate the symptoms in a clinical setting. Furthermore, the clinical diagnoses were based on neuropsychological assessments and biomarker evidence for AD pathophysiological processes [36, 44]. Thus, their MCI due to AD cases can be considered to be “truly” on the Alzheimer’s continuum. Our aMCI diagnosis was solely based on the “core clinical criteria” by Albert et al. [36] as we did not have biomarker information. There is also a difference in the control group between studies. In the study by Teuber-Hanselmann et al., the control group was very heterogeneous consisting of healthy participants and patients with headache, psychiatric diseases, or Parkinson’s disease. Additionally, storage duration of the samples differed considerably in both studies, with 50% or more storage time in the present study. Thus, both studies cannot be compared directly. However, both studies show that the diagnostic accuracy of blood KLK8 is very high.

The diagnostic accuracy of KLK8 is not only important for an early AD diagnosis, but based on its therapeutic potential shown in transgenic mice [19], possibly also relevant for patient stratification in therapeutic settings. So far, clinical trials using Aβ and tau as therapeutic targets have led to contradictory and sobering observations [45], thus making the need for new players even direr. Although different KLK8 substrates, e.g., ephrin receptor B2 (EPHB2) [46], fibronectin [47], neuregulin-1 [48], and the neural cell adhesion molecule L1 (L1CAM) [49], have been long known to be directly involved in the pathophysiology of AD, it was not until recently that the role of KLK8 in the context of AD was recognized. Our lab was the first to demonstrate elevated cerebral KLK8 levels in the very early stages of AD disease, i.e., in AD patients with the onset of the first Aβ plaques and in transgenic mice even before the onset of Aβ pathology [19]. Moreover, several aspects of AD pathology could be alleviated by antibody-mediated inhibition of KLK8 [19, 21] or the genetic knockdown of KLK8 in transgenic mice [22]. Interestingly, single nucleotide polymorphisms (SNPs) in KLK8 [50] similar to CD33 [51], TOMM40 [52], and APOE [53] are all located in the same chromosomal region 19q13 which apparently is strongly associated with AD risk. The present study alongside with the aforementioned findings adds now another piece evidence to prove a role for KLK8 in the emergence of AD.

### Strengths

Our study has several strengths. Our cases and controls are derived from a large, randomly selected population-based sample with high quality of data collection and processing which was confirmed by external certification of the HNR study. All cases and controls were free of other major diseases and in the normal range of inflammatory parameters. Furthermore, we were able to perform an excellent matching of cases and controls by sex and age. Unlike in the previous case-control study [23], collection of blood was performed in one central reference laboratory and the storage duration of samples was very similar.

### Limitations

The major limitation is that our aMCI diagnosis is not based on AD biomarker information. Thus, we cannot state whether our aMCI participants are on the “Alzheimer’s continuum” as defined by Jack et al. [3]. However, we have excluded participants with Parkinson’s disease, stroke, and tumor disease to exclude cognitive impairment due to those diseases. Nonetheless, a confounding effect of an unknown comorbidity or premedication cannot be entirely excluded. A further limitation is the size of the finally analyzed population. Our study was primary designed as a cardiovascular health study on myocardial infarction with long follow-up times between examinations (every 5 years) and a relatively young cohort of participants (45–75 years at baseline). Thus, we were not able to include a group of participants with incident AD to this analysis at the current point. Currently our dementia endpoint committee is gathering data from follow-up questionnaires and medical reports to identify those individuals who progressed to AD in the further course. Blood KLK8 levels of these participants will be then determined at T2 (>5 years before AD onset) and T1 (>10 years before AD onset) to verify KLK8 potential in predicting presymptomatic AD.

## Conclusions

Our study is the first population-based case-control study to demonstrate the capability of KLK8 as a blood biomarker for early diagnosis of AD. A 500 pg/ml increase in blood KLK8 was associated with an almost threefold increased chance of an aMCI diagnosis compared to cognitively unimpaired participants. The diagnostic performance of KLK8 in blood for aMCI was very good with an AUC of 0.92. Larger validation studies in a longitudinal design are now warranted.

## Supplementary Information

**Additional file 1.** Supplementary materials

## Data Availability

The corresponding author has full access to all data in the study and final responsibility for the submission of the article for publication. Due to data security reasons (i.e., data contain potentially participant identifying information), the HNR study does not allow sharing data as a public use file. Data requests can also be addressed to recall@uk-essen.de.

## Abbreviations

KLK8: Kallikrein-8
AD: Alzheimer’s disease
aMCI: Amnestic mild cognitive impairment
CU: Cognitive unimpaired
T1: Five years after baseline
T2: Ten years after baseline
OR: Odds ratios
95%CI: 95% confidence intervals
ROC: Receiver operating characteristic
AUC: Area under the curve
SD: Standard deviation
Aβ: Beta-amyloid
CSF: Cerebrospinal fluid
MCI: Mild cognitive impairment
HNR study: Heinz Nixdorf Recall study
Recall: Risk Factors, Evaluation of Coronary Calcification, and Lifestyle
SCD: Subjective cognitive decline
naMCI: Non-amnestic mild cognitive impairment
ADL: Activities of daily living
hsCRP: High-sensitivity C-reactive protein
ELISA: Enzyme-linked immunosorbent assay
TMT: Trail Making Test
WHO: World Health Organization
BMI: Body mass index
ISCED: International Standard Classification of Education
APOE ε4: Apolipoprotein E ε4
EPHB2: Ephrin receptor B2
L1CAM: Neural cell adhesion molecule L1
SNPs: Single nucleotide polymorphisms
