# Supplementary material for: Is kallikrein-8 a blood biomarker for detecting amnestic mild cognitive impairment? Results of the population-based Heinz Nixdorf Recall study
Source: Alzheimers Res Ther. 2021 Dec 20;13:202. doi: 10.1186/s13195-021-00945-x (PMC8690879; doi:10.1186/s13195-021-00945-x)
Supplement: Supplementary file 1 — Additional file 1. Supplementary materials [file 13195_2021_945_MOESM1_ESM.docx]

**Supplementary material**

1. **Matched Case-Control Power Analysis**

**Numeric Results**

**Cases Controls Per Odds Ratio Probability Correlation**

**Power (N) Case (M) (OR) Exposed (P0) (Phi) Alpha Beta**

0,80000 40 1 10,09 0,05000 0,20000 0,05000 0,20000

0,80000 40 2 7,03 0,05000 0,20000 0,05000 0,20000

0,80000 40 3 6,10 0,05000 0,20000 0,05000 0,20000

0,80000 40 1 6,46 0,10000 0,20000 0,05000 0,20000

0,80000 40 2 4,84 0,10000 0,20000 0,05000 0,20000

0,80000 40 3 4,30 0,10000 0,20000 0,05000 0,20000

0,80000 40 1 5,31 0,15000 0,20000 0,05000 0,20000

0,80000 40 2 4,09 0,15000 0,20000 0,05000 0,20000

0,80000 40 3 3,67 0,15000 0,20000 0,05000 0,20000

0,80000 40 1 4,77 0,20000 0,20000 0,05000 0,20000

0,80000 40 2 3,72 0,20000 0,20000 0,05000 0,20000

0,80000 40 3 3,36 0,20000 0,20000 0,05000 0,20000

0,80000 40 1 4,48 0,25000 0,20000 0,05000 0,20000

0,80000 40 2 3,51 0,25000 0,20000 0,05000 0,20000

0,80000 40 3 3,19 0,25000 0,20000 0,05000 0,20000

0,80000 40 1 4,33 0,30000 0,20000 0,05000 0,20000

0,80000 40 2 3,40 0,30000 0,20000 0,05000 0,20000

0,80000 40 3 3,09 0,30000 0,20000 0,05000 0,20000

**1a. References**

'Power Calculations for Matched Case-Control Studies', Biometrics, Volume 44, pages 1157-1168.

**1b. Report Definitions**

Power is the probability of rejecting a false null hypothesis.

N is the size of the sample drawn from the treatment (case) group.

M is the number of matching control patients drawn for each case patient.

OR is the odds ratio of for subjects exposed to the risk factor.

P0 is the probability of exposure among sampled control patients.

Phi is the correlation of exposure between matched individuals.

Alpha is the probability of rejecting a true null hypothesis.

Beta is the probability of accepting a false null hypothesis.

**1c. Summary Statements**

In a matched case-control study, the probability of exposure among sampled control patients is

0,05000 and the correlation coefficient for exposure between matched case and control patents

is 0,20000. A sample of 40 case patients is obtained. For each case patient, a matching sample

of 1 control patient(s) is also obtained. This sample of 80 patients achieves 80% power to

detect an odds ratio of 10,09 versus the alternative of equal odds using a Chi-Square test with

a 0,05000 significance level.

**1d. Chart Section**

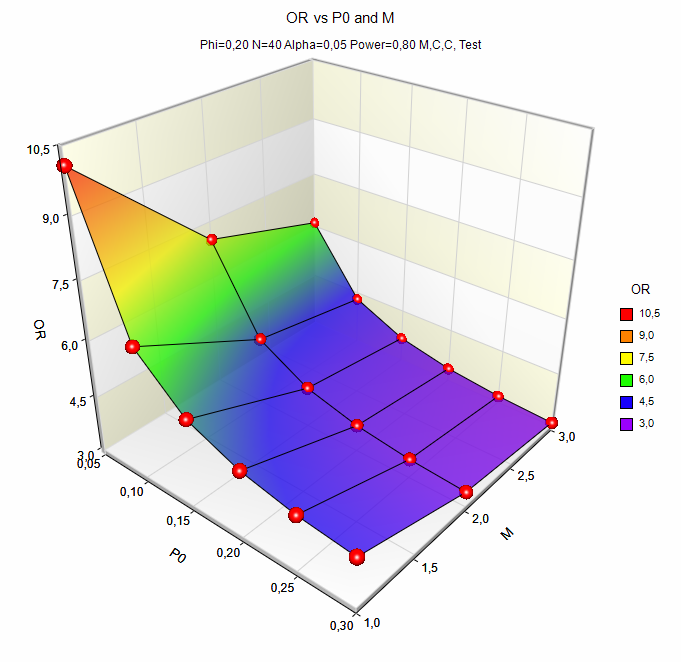


1. **Supplementary tables and figures**

**Supplementary figure 1.** Agreement between the two KLK8 measurements. Fit plot. KLK8 in pg/ml.

**Supplementary figure 2.** Agreement between the two KLK8 measurements. Bland Altman plot. KLK8 in pg/ml.

**Supplementary table 1.** Association between KLK8 and cognitive status (aMCI vs. CU) after excluding participants with depression at T2.

| **n=103** | **OR** | **95%CI** | | **OR** | **95%CI** | |
| --- | --- | --- | --- | --- | --- | --- |
| **per 500 pg/ml KLK8** | 1.210 | 0.873 | 1.678 | 3.132 | 1.089 | 9.010 |
| **experimenter 2 vs. 3*** |  |  |  | 0.026 | <0.001 | 0.691 |
| **experimenter 1 vs. 3*** |  |  |  | 5.658 | 0.830 | 38.591 |
| **freezing duration, years** |  |  |  | 0.920 | 0.825 | 1.026 |

Legend: aMCI, amnestic mild cognitive impairment; CU, cognitive unimpaired; T2, ten-year follow-up; OR, odds ratio; 95%CI, 95%-confidence interval; KLK8, kallikrein 8. Age and sex are taken into account by matching for them. *We adjusted for experimenter as a proxy for the inter-assay variability.

**Supplementary table 2.** Association between KLK8 and cognitive status (aMCI vs. CU) using T1 criteria at T2.

| **n=105** | **OR** | **95%CI** | | **OR** | **95%CI** | |
| --- | --- | --- | --- | --- | --- | --- |
| **per 500 pg/ml KLK8** | 1.087 | 0.802 | 1.472 | 2.530 | 1.021 | 6.271 |
| **experimenter 2 vs. 3*** |  |  |  | 0.028 | 0.002 | 0.505 |
| **experimenter 1 vs. 3*** |  |  |  | 4.882 | 0.732 | 32.565 |
| **freezing duration, years** |  |  |  | 0.927 | 0.839 | 1.023 |

Legend: aMCI, amnestic mild cognitive impairment; CU, cognitive unimpaired; T1, five-year follow-up; T2, ten-year follow-up; OR, odds ratio; 95%CI, 95%-confidence interval; KLK8, kallikrein 8. Age and sex are taken into account by matching for them. *We adjusted for experimenter as a proxy for the inter-assay variability.

**Supplementary figure 3.** Diagnostic performance of KLK8 to discriminate between participants with amnestic mild cognitive impairment and cognitive unimpaired according to receiver operating characteristic (ROC) analyses, unadjusted.
